# Personal

**Published:** 1895-02

**Authors:** 


### Personal.

Dr. Henry C. Buswell, of Buffalo, has recently returned from a two years' visit in Europe, where he has been in attendance upon the clinics in Vienna, Berlin, Heidelberg, Paris and London. Dr. Buswell is adjunct professor of the principles and practice of medicine in Niagara University Medical College, and his colleagues, professorial and professional, united in tendering him a banquet at the Tifft House in this city, on Saturday evening, December 29, 1894. Dr. F. S. Crego was toastmaster, and Dr. John Cronyn made an address of welcome. Dr. Buswell, in responding, thanked those present for the flattering reception they had given him. He reviewed some of the principal clinics, in which he participated, told of the use of the new diphtheritic remedy and the treatment of typhoid fever by the use of the bacillus.

Other toasts were The Medical Department of Niagara University, by Dr. Lothrop; Foreign Universities, by Dr. Pryor; The Academy of Medicine, by Dr. Van Peyma; State Hospitals, by Dr. A. W. Hurd; Canadian Universities, by Dr. H. E. Hayd; Advance in Sanitary Science, by Dr. Ernest Wende, and American Universities, by Dr. H. A. Weed. Letters of regret were read from Drs. Mynter, Tremaine, Frederick, Mickle, Krauss, Renner, Martin and Hunt.

Dr. Percy Bryant has been appointed first assistant physician in the Buffalo State Hospital, to succeed Dr. Arthur W. Hurd, promoted superintendent. This appointment is made from the State civil-service list, and Dr. Bryant is in every way well fitted for and deserving of the place. He is a member of several medical organizations and a companion (hereditary) of the Military Order of the Loyal Legion of the United States, in affiliation with the New York commandery.

Drs. William G. Bissell and Thomas B. Carpenter have been appointed, respectively, bacteriologist and assistant bacteriologist to the health department of Buffalo, subject to the civil-service rules. Their appointments are regarded as especially satisfactory, as these gentlemen have each had special training in the field of bacteriology.

---

Dr. L. S. McMurtry, of Louisville, Ky., has recently occupied his new residence at No. 12 St. James's Court. His offices, however, will be continued at 231 West Chestnut street, where he can be consulted on Mondays, Wednesdays and Fridays between the hours of 11 and 1, but on other days only by appointment.

---

Dr. Daniel Lewis, of 249 Madison avenue, New York, has changed his consultation hours as follows: Daily from 10 to 1 and by appointment. He has also arranged for the reception of his operative cases in a private hospital near his office, where every convenience is assured to the patients.

---

Dr. G. R. Trowbridge who, for several years was assistant physician in the State Hospital for the Insane at Danville, Pa., has returned to Buffalo and established his offices at 1331 Main street. His numerous friends here will welcome him to his old home.

---

Dr. A. L. Benedict, of Buffalo, read a paper on the Purely Physical Examination of the Digestive Organs at the annual meeting of the Medical Society of the County of Ontario, held at Canandaigua, N. Y., Tuesday, January 8, 1895.

---

Dr. Joseph D. Bryant recently was elected president of the New York Academy of Medicine, vice Dr. D. B. St. John Roosa, whose term had expired. If Dr. Roosa must needs have a successor in this office, Dr. Bryant is in every way worthy of the honor.

---

Dr. A. H. Cordier, of Kansas City, Mo., has recently published in the *Journal of the American Medical Association* a paper detailing fifty consecutive intra-peritoneal operations with but one death.

